# Stable laboratory culture system for the ctenophore *Mnemiopsis leidyi*

**DOI:** 10.1007/978-1-0716-3642-8_4

**Published:** 2024-01-01

**Authors:** Joan J. Soto-Angel, Eva-Lena Nordmann, Daniela Sturm, Maria Sachkova, Kevin Pang, Pawel Burkhardt

**Affiliations:** 1 Michael Sars Centre, University of Bergen, Norway; 2 University of Plymouth, United Kingdom

**Keywords:** Breeding, Ctenophora, comb jelly, cultivation, husbandry, protocol, sea walnut

## Abstract

Ctenophores are marine organisms attracting significant attention from evolutionary, molecular biology and ecological research. Here we describe an easy and affordable set-up to maintain a stable culture of the ctenophore *Mnemiopsis leidyi*. The challenging delicacy of the lobate ctenophores can be met by monitoring the water quality, providing the right nutrition, and adapting the handling and tank set-up to their fragile gelatinous body plan. Following this protocol allows stable laboratory lines, a continuous supply of embryos for molecular biological studies, and independence from population responses to environmental fluctuations.

## 1 Introduction

Ctenophores (comb jellies) are candidates for being one of the earliest extant lineage of animals and thus hold a key phylogenetic position to study the origin of animals, their nervous system and cell types [1–8]. Reconstructions of ancient gene linkages inferred from high-quality genomes of a ctenophore, two sponges and three unicellular relatives of animals tipped the scales on ctenophores as the most probable sister group to all other metazoans lineages [9]. Moreover, the high invasive potential and predatory impact of some ctenophore species have led to increasing attention of this phylum in marine ecosystem studies [10]. Although ctenophores have been reported already in 1671 [11], the first species were described in the late seventeen hundreds [12–14], while the formal description of the taxon Ctenophora was in 1829 [15, 16]. *Mnemiopsis leidyi* was described in 1865 [17], ca. 200 years after the first reports of ctenophore species.

Ctenophore cultivation may be challenging since most species are extremely fragile and sensitive to abrupt changes in environmental conditions. Indeed, conventional aquaria are unsatisfactory for mid- to long-term ctenophore cultivation (e.g. [18]). This might have been a major reason for most studies up until recently to resort to wild-caught animals and their direct offspring, instead of keeping a steady culture in the laboratory. Given these methodological constraints, pioneer cultivation attempts aimed to develop new culture methods provided with gentle but constant water-flow and tanks without sharp edges that helped to minimize animal damage [19–21]. These systems were later modified and are currently successfully used in many jellyfish exhibitions in public aquariums worldwide [22, 23]. Subsequent contributions focused on the trophic and reproductive biology of selected ctenophore species, making available the first culture procedures [24, 25]. In the last years, detailed protocols have been made available, including spawning and embryo collection [26, 27], a new spawning and cydippid larvae rearing method [28], a laboratory aquaculture system for behavior characterization [29], and protocols to maintain and isolate *M. leidyi* in vitro cell cultures [30], and to study whole body regeneration [31]. Those contributions mostly focused on a few aspects of ctenophore cultivation. Very recently, comprehensive protocols covering all aspects of long-term ctenophore husbandry in the lobate ctenophores *M. leidyi* [32] and *Bolinopsis mikado* [33] have been published. However, those setups needed elaborated recirculating systems with the concomitant high amount of seawater (and other resources) required. The aim of this contribution is to pave the way for a successful *M. leidyi* husbandry by providing an alternative culture system that is simple, accessible and efficient.

The lobate ctenophore *Mnemiopsis leidyi* is an emerging model system for molecular biology studies. Its genome [2], single cell transcriptome [34], temporal developmental expression profiles [35], methylome [36] as well as molecular biological techniques such as in situ hybridization [37, 38], immunostaining [38, 39], gene knockdown by morpholinos [40, 41] and gene manipulations by CRISPR-Cas9 [42] recently became available. A stable culture system for *M. leidyi* enables opportunities for molecular biology, behavior studies and evolutionary research [43–46].

Following the pioneer work of Baker and Reeve [25], Pang and Martindale [26], Salinas-Saavedra and Martindale [27], and the more recent Patry et al. [28], Ramon-Mateu et al. [31], Presnell et al. [32], and Ikeda et al. [33] we here report an affordable set-up and step-by-step protocol for culturing the ctenophore *M. leidyi*. The first generation was originally sampled from Kristineberg, Sweden, Baltic Sea, and corresponds to the same population as the specimen of the recently published whole-organism single-cell RNA sequencing study [34]. Our protocol covers all aspects of a long-term, multigenerational *M. leidyi* culture, including facility set-up, sampling, daily routine, handling, breeding, hatchery and nursery. Following this protocol, we have maintained a culture of *M. leidyi* with thousands of specimens belonging to twenty-one generations. In addition, the adult setup and nursery methods described here have been successfully assayed and can be equally applied to the common northern comb jelly *Bolinopsis infundibulum*, a species which laboratory culture had not been described until now.

## 2 Materials

### 2.1 Tank setup, transfer and nursery

- 6 L transparent seawater tanks (CAT# RFSCW8, CAMBRO®)
- 15 L transparent seawater tanks (CAT# RFSCW18, CAMBRO®)
- Rectangular aquarium, at least 50 cm wide
- Aquarium water chiller
- Water pump
- Gear motor(s) 15 RPM
- 25 ml serological pipettes (e.g. CAT# 734-0343, VWR® Europe)
- Flexible silicone hose, 5-6 mm diameter
- Light tubes (neutral white to sunlight, 4500-5500 K)
- Plug-in timer
- Transfer pipettes 3.5 ml (e.g. CAT# 86.1171 Sarstedt®)
- 60 mL crystalizing dish without spout (e.g. CAT# 216-1862, VWR® Europe)
- 300 mL crystalizing dish with spout (e.g. CAT# 216-1815, VWR® Europe)
- 90 mm tissue culture dish (e.g. CAT# 734-2795, VWR® Europe)

**Fig. 1:**
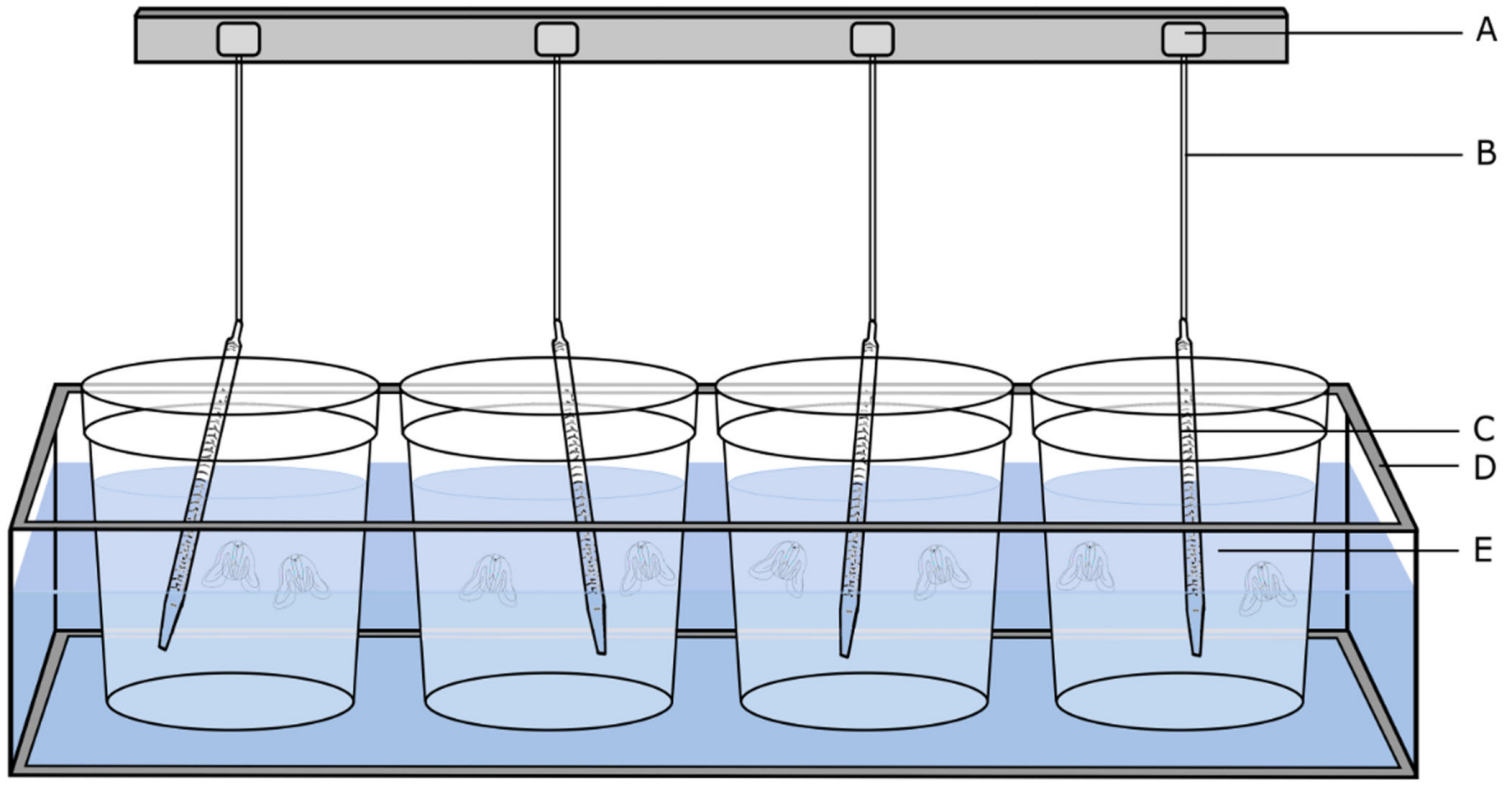
Tank setup for *Mnemiopsis leidyi* culture. (**A**) gear motor, (**B**) flexible silicone hose, (**C**) 25 ml serological pipette, (**D**) rectangular aquarium, (**E**) seawater tank. Water in the rectangular aquarium acts as water bath and is thermostated through an aquarium water chiller (not shown).

The culture system consists of transparent, cylindrical, open at the top, 6-15 L, seawater tanks, which are partially submerged in a larger rectangular aquarium (Fig. 1). The latter is filled with fresh water and refrigerated through an aquarium water chiller coupled to a pump. The flow rate should be in accordance with the water chiller requirements provided by the manufacturer (see Note 1). The culture tanks are therefore refrigerated by water bath (see Note 2) and kept at constant temperature, adjustable in a range between 14-18 °C (see section 2.2 on water quality for other water parameters). Circular current flow is generated by a rotating and oscillating 25 ml serological pipettes (see Note 3), ca. 350 mm long and 13 mm thick. The motion pipettes are connected through a flexible silicone hose to a small gear motor running constantly at 15 RPM. Light tubes (4500-5500 K) are mounted beneath the transparent bottom of the rectangular aquarium and their switching on and off is controlled by a plug-in timer, with a regime of 12 hours light and 12 hours dark. See section 3.1 for transfer and section 3.2.3 for details on nursery.

### 2.2 Water quality

- Mechanical filter cylinders: Mesh 20, 10 and 1 µm
- Active charcoal filter, ca. 0.45 µm
- Ultraviolet lamps and irradiation cylinder/panel
- Portable pH and conductivity meter (e.g. "WTW™ ProfiLine™ pH/Cond 3320; CAT# 15206778, FisherScientific®).
- Carboys 20 L (e.g. CAT# 216-1700, VWR® Europe)

Purified Sea Water (PSW) used both for the main *Mnemiopsis* culture and auxiliary cultures is obtained by filtrating seawater through mechanical filters (20, 10 and 1 µm mesh-size, in that sequence), later purified through an active charcoal filter (0.45 µm mesh-size), and finally UV treated. Water outlet and all recipients used must be kept clean (see Notes 4-6). Water parameters (temperature, pH and salinity) are measured before water usage (see Note 7). Optimum values for temperature between 14-18º C, salinity between 28-30‰, and pH between 7.8-8.1.

### 2.3 Auxiliary cultures

- Living *Brachionus* (rotifers)
- Concentrated algae (e.g. *Nannochloropsis*-based products, RG complete™ or similar).
- *Artemia* cysts
- *Artemia* hatcher or equivalent
- 6 L seawater transparent tanks (CAT# RFSCW8, CAMWEAR®, CAMBRO®)
- Lid for 6 L seawater tanks (CAT# RFSCWC6, CAMWEAR®, CAMBRO®)
- Air pumps or equivalents
- Frozen copepods 0.7-1.8 mm
- Centrifuge tubes 50 ml (e.g. CAT# 62.547.254, Sarstedt®)
- Microcentrifuge tubes 1.5 ml (e.g CAT# 211-2164, VWR® Europe)
- 10 ml serological pipettes (e.g CAT# 7342-0352, VWR® Europe)
- Transfer pipettes 3.5 ml (e.g. CAT# 86.1171 Sarstedt®)
- 100 ml plastic beaker (e.g CAT# 213-1623, VWR® Europe)
- 2 L beaker with spout (e.g CAT# 213-3404, VWR® Europe)
- Flexible silicone hose, 5-6 mm diameter
- High-capacity filters (200-300 ml) 70 µm, 150 µm and 500 µm

Ctenophores are fed daily with a variety of prey items, including at least two of the following: *Brachionus* sp. (Rotifera), freshly hatched *Artemia* (brine shrimp) (instar I stage), *Artemia* 24 hours post-hatching (hph) (instar II stage), and copepods. *Brachionus* permanent culture is kept in 6 L tanks with lid and constant aeration. Freshly hatched *Artemia* nauplii are used directly, with no supplements. *Artemia* nauplii ca. 24 hph are enriched with living algae in order to increase its nutritional profile. This step does not require keeping algae cultures, as concentrated algae (e.g. RG complete) that are used to feed *Brachionus* can be conveniently used to feed *Artemia* 24 hph as well.

## 3 Methods

### 3.1 Acquisition and handling

Regardless of whether the ctenophores were sampled in the wild or obtained from a previous culture, handling procedures require extreme care, as the animals are particularly fragile and can easily be injured (see Note 7). In order to relocate a specimen from the sea to a container or from a container to another, proceed as described in section 3.1.2. Note that the smaller individuals, specifically the cydippid larvae are more robust than the lobed, larger individuals (see Note 8). Laboratory gloves are generally not recommended for the culture, since even non-powdered gloves seem to leave trace residues in the water. If gloves are needed, we recommend to thoroughly rinse them with PSW before they are used.

#### 3.1.1 Sampling

Some shallow-water ctenophore species (e.g. *Pleurobrachia pileus, Beroe ovata, Mnemiopsis leidyi* and *Bolinopsis infundibulum*) can be easily sampled on board of a vessel that allows reaching specimens by hand. Alternatively, most species can be obtained by snorkelling or by underwater SCUBA diving when appropriate. *Mnemiopsis leidyi* is a coastal blooming species, relatively easy to spot and sampled from the shore, both in natural locations and in harbours all around its habitat. *Mnemiopsis leidyi* is widely distributed, forming native populations in western Atlantic, and introduced populations in the North Sea, the Baltic Sea and the Mediterranean Sea [47]. Previous mentioned methods are the most adequate to sample *M. leidyi*. However, the use of nets is necessary when its abundance is low. Net sampling is generally a stressing procedure for the animals that often results in mechanically injured specimens. If nets are strictly required, the use of a relatively large mesh size (i.e. 800 µm), slow towing speed (between 1.5 and 2 knots, i.e. 0.75-1 m/s), and large (e.g. 3 L) closed, non-filtering, cod-end can minimize severe damage. Ensure to keep the sampled ctenophores first in the original seawater (you may want to take water with you) (see Note 7), and only gradually acclimate the sampled animals to the new laboratory conditions, keeping water parameters as close as possible to the original water source.

#### 3.1.2 Transferring adult ctenophores

Whether the adult ctenophores are directly obtained from the wild or another culture, proceed as follows (for larvae transfer, see section 3.2.3).

- Ensure that the receiving tank is already available and filled with appropriate water (seawater from the sampling site in case of sampling, fresh PSW in case of culture), at a similar temperature, salinity and pH than the original one.
- Select an appropriate transfer container (e.g. a 1 L jar/bottle for sampling or a 50-100 ml beaker for transferring), preferably rounded and without any sharp edges or spout, with an opening large enough to maneuver easily with the specimen (e.g. 60 mL crystalizing dish without spout).
- Rinse your hands/laboratory gloves thoroughly with warm fresh water and dry them with a cloth that leaves no residues.
- Rinse the transfer container with PSW at similar temperature (± 3º C) to the one where the animals are staying.
- Slowly submerge the transfer container into the water avoiding turbulences or abrupt water intake.
- Let the transfer container fill completely with water before continuing (no large bubbles should remain), and move it towards the specimen to be transferred (either laterally or vertically depending on the depth and position of the animal). Avoid touching, moving, turning or pushing the animals neither with your hands nor with the transfer container, especially with the edges. If needed, use the transfer container to create a slow current to move the ctenophore to the desired position.
- Once the specimen is completely within the transfer container, and as long as it is in a safe position to avoid damaging the lobes, lift the transfer container as vertically as possible and take it out from the water carefully.
- If needed, remove as much water as possible from the transfer container (see Note 9). Healthy *M. leidyi* tolerate slight levels of compression between the water surface and the bottom of the container (see below).
- If the beaker with the animal is placed on a not-completely clean surface, make sure to clean it before proceeding.
- Introduce the transfer container with the animal into the new tank, avoid turbulences and/or rapid water flow. This can be done by ensuring that the water level between the transfer container and the receiving tank are aligned before submerging. Simultaneously turn and lift the transfer container carefully, slowly releasing both the water and the animal into the receiving container (see Note 9). Avoid pouring the animals or making them slide down through the surface of the transfer container.
- Observe the recently transferred animal. If there are some signs of damage, proceed as described in Note 10.

### 3.2 The culture

For a description of tank setup and water quality see section 2.1 and section 2.2 respectively. The system presented here has allowed us to successfully keep an affordable long-term *Mnemiopsis* culture. Routines and protocols described are specifically designed to minimize the time and costs invested, maximize the production, and allow an easy access and collection of the animals. Their implementation has allowed us to get several thousands of individuals belonging to eleven generations from an original set of 5 individuals, and to get several thousands of viable eggs and larvae per week. Several individuals have been kept to date for more than 3.5 years. Occasional loss of some individuals is compensated by the rapid growth rate and high production of larvae. Thus, the described culture system allows producing a considerable surplus of specimens available for experimentation.

**Fig. 2:**
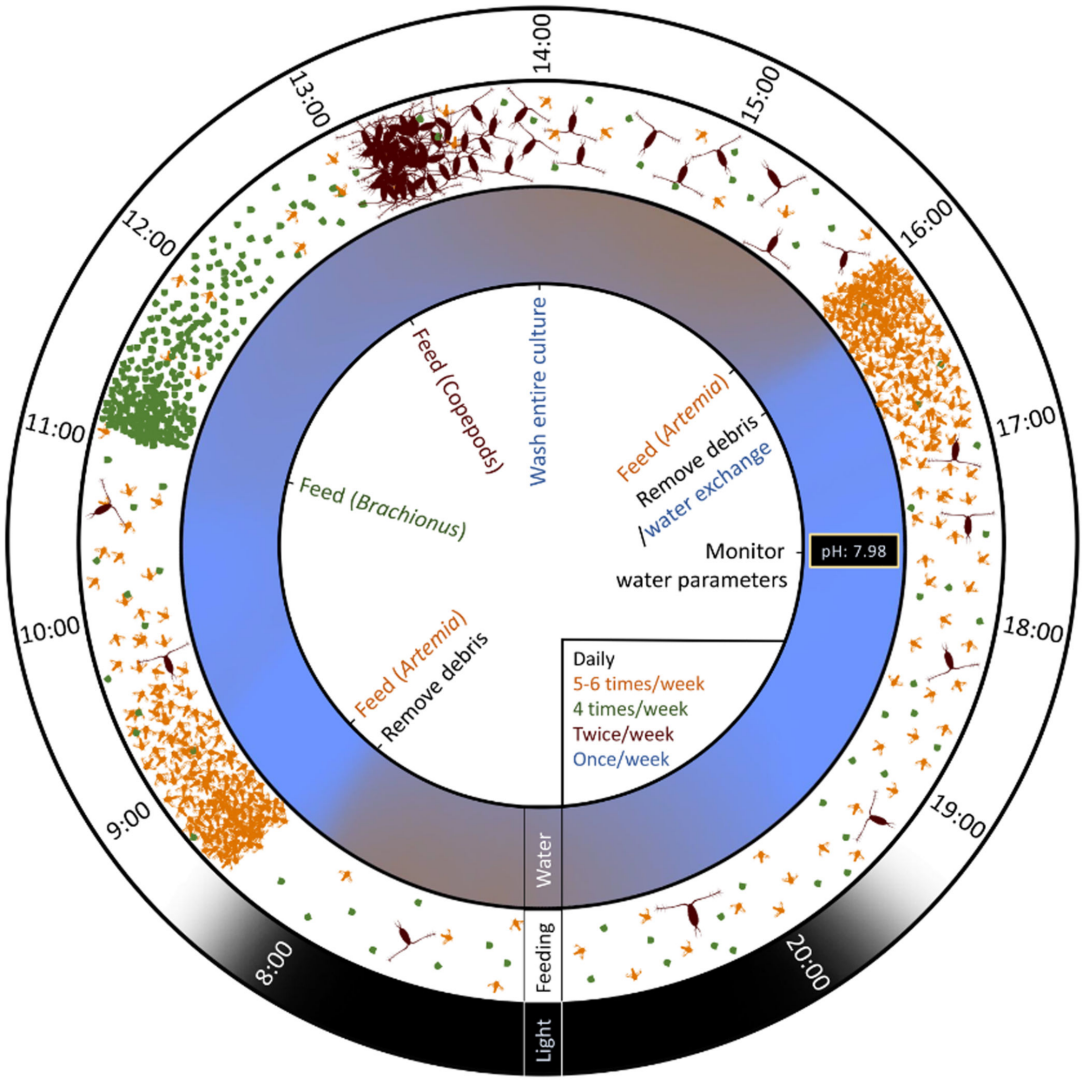
Daily feeding and washing regimes for *Mnemiopsis leidyi* culture.

The recommended weekly plan (Table 1) requires a basic maintenance that includes feeding (3-4 times/day, 5 days/week), cleaning and washing (debris removed daily, weekly partial water exchange and weekly full water exchange) and water quality monitoring when appropriate. Therefore, five consecutive days per week are required once the culture is established. An overview of the daily routines is illustrated in Fig. 2.

Ctenophore density in the tanks can vary depending on the needs and the space availability, with maximum values adjusted according to their average size. Ideally, density of medium to large adult ctenophores (>12 mm aboral-oral length excluding lobes) should not exceed one specimen/L. Smaller individuals and larvae can be kept at much higher densities (see Note 11).

#### 3.2.1 Rearing

##### 3.2.1.1 Feeding

An overview of the feeding regime is described in section 2.3 and illustrated in Fig. 2. *Mnemiopsis* specimens should always have some prey available in the tank, but overfeeding must be avoided, as the uneaten food will deteriorate water quality. An optimal strategy consists of splitting daily feeding amounts into several doses. Indicative amounts of food per ctenophore are given for each food source. In any case, we recommend to individually assess different amounts in order to find the most suitable ones for a given culture condition.

###### 3.2.1.1.1 Brachionus

As mentioned above, the rotifer *Brachionus* (150-400 µm size) is the only permanent auxiliary culture needed, since it is simple to maintain and suitable for feeding all *Mnemiopsis* life stages, and particularly convenient for the earliest ones (see section 3.2.3). The culture is kept in 6 L tanks at room temperature (18-20 ºC), with a lid and a central opening for a 10 ml serological pipette providing permanent aeration. The food for *Brachionus* consists of concentrated algae (see materials section). Overfeeding must be prevented to avoid population crash (see Note 12). In order to maximize production, rotifers are fed daily from Monday to Friday. Each *Brachionus* tank is washed completely once a week (see protocol below). Under these conditions, optimum population densities (ca. 200.000-400.000 individuals/L) are obtained just a few days after the start of the culture. Subsequently, each *Brachionus* culture tank is harvested 4-5 times/week, for approximately one fifth of the total volume for each harvest. As a general guide, 2000-4000 rotifers per adult *Mnemiopsis* per day (4 times/week) is an adequate amount for the culture conditions described, but note that this will greatly depend on factors such as ctenophore size, fitness and water quality parameters. Cydippid larvae require between 50-200 rotifers per larva per day (4 times/week) depending on the size. Transitional stages I and II require intermediate values between the ones mentioned for larvae and adults. Therefore 1 L of *Brachionus* culture in optimum densities potentially allows to feed one of the following combinations (for 1 day): 1) 150 adults, 2) 1500 cydippid larvae, 3) 100 adults + 500 cydippid larvae or 4) 50 adults + 1000 cydippid larvae.

Harvest *Brachionus* to feed the ctenophores (Note 13):

- Turn off aeration by removing the pipette and let algae aggregates sink down.
- After approximately 10 minutes, take the required amount of *Brachionus* culture with a 1 L beaker, with an upper limit of one fifth the total culture tank volume (Note 14).
- Filter and rinse gently through a 70 µm mesh above the sink with 2 L of PSW in order to remove the remaining algae (Note 15).
- In order to maximize the algae removal, leave the filter in a 1 L beaker with 400 ml of PSW, wait for 10-20 minutes and repeat the previous step.
- Fill up the used *Brachionus* culture tank with PSW at room temperature, with the same water volume that has been taken out.
- Place back the aeration pipette and use it to mix the culture.
- Check that the aeration pipette reaches the bottom of the tank, and that air flow is adequate.
- Feed the Ctenophores with *Brachionus* using a new transfer pipette previously washed (in and out surfaces) with PSW, 1 dose/day (see above for indicative amounts).
  Washing the *Brachionus* cultures:
- Prepare the number of 6 L tank(s) needed with PSW at room temperature.
- Turn off aeration from the old tank by removing the pipette and let the algae sink down.
- After approximately 10 minutes, filter the complete *Brachionus* culture through a 70 µm mesh, avoiding the most basal section where the algae aggregates are deposited (see Note 16).
- Rinse the *Brachionus* in the filter with an additional 1 L of PSW (see Note 15).
- If bubbles (i.e. dead *Brachionus* and debris) are present in large amounts, remove them with the aid of a transfer pipette.
- Wash off the *Brachionus* from the 70 µm mesh into the new tank with PSW.
- Install a new 10 ml serological pipette and set them for intermediate levels of aeration.
- Check that the aeration pipette reaches the bottom of the tank, and that air flow is adequate.
  Feeding *Brachionus*:
- Add approximately 1-2 ml of concentrated living algae (refrigerated) per every 5 L of *Brachionus* culture to a 100 ml plastic beaker with 30-40 ml of PSW and mix with a transfer pipette. If the following days the *Brachionus* culture will not be fed, use twice the indicated amount of concentrated algae (but see Note 12).
- Feed *Brachionus* culture with the prepared algae using a transfer pipette. Add an equivalent amount to each tank. Should differences in color density be observed, add a larger amount to the less greenish (more transparent or more brownish) tanks.
- Mix thoroughly the water in the bucket using the aeration pipette.
- Check that the aeration pipette reaches the bottom of the tank and that air flow is adequate.

###### 3.2.1.1.2 Artemia

*Artemia* (brine shrimp) is a widely used food source in aquaculture. A great variety of protocols for cysts hatching and nauplii larvae harvesting are easily accessible and normally provided by the manufacturer. Therefore, only some considerations regarding the use of *Artemia* for *Mnemiopsis* culture are provided:

- Special care must be taken when harvesting and rinsing *Artemia* to wash off the majority of the accompanying heterotrophic bacteria and the unhatched cysts.
- Recently hatched *Artemia* (Instar I stage, 350-550 µm, 18-22 hours after cysts incubation at 24-26 ºC, not feeding) is the most nutritious form when the nauplii are not fed, and is therefore preferred over older nauplii when no enrichment is performed.
- Instar II stage (450-650 µm, 26-30 hours after incubation at 24-26 ºC, feeding) are metanauplii that have already consumed most of their reserves and are ready to ingest food. The enrichment at this stage through feeding with algae rich in Highly Unsaturated Fatty Acids (HUFA) has shown multiple benefits in aquaculture [48, 49], and *M. leidyi* spawning seems to be boosted when enriched nauplii are used as food source. For this purpose, concentrated algae such as the widely used *Nannochloropsis*-based solutions or RG complete (*Brachionus* food) can be employed. The enrichment process requires a few drops of concentrated algae and takes a minimum of 1 hour (ideally 3-4 hours) since the algae mixture is added.
- Most attempts of feeding *M. leidyi* with adult *Artemia* resulted unsatisfactory, probably due to the high swimming speed of the later. We therefore do not recommend using *Artemia* older than 48 hph.
- Before feeding the ctenophores with either Instar I or II stages, the nauplii should be rinsed thoroughly with PSW through a 150 µm mesh filter.
- A larger number of small doses of *Artemia* nauplii is preferred over a single large dose. This will allow ctenophores to constantly have available prey, reduce debris, and will keep the nauplii in their optimal nutritional profile just before being added to the ctenophore culture. For doses later in the day, keep Instar I refrigerated at 4-5 ºC.
- As an indicative value, 200-1000 *Artemia* nauplii per adult *Mnemiopsis* and day (5 times/week) is an adequate amount for the culture conditions described, but note that this will greatly depend on factors such as ctenophore size, fitness and water quality parameters.
- At its maximum growth rate, *M. leidyi* can start feeding *Artemia* when they are 10 days old (but see 3.2.3).

###### 3.2.1.1.3 Copepods

Copepods are a convenient prey for *M. leidyi*, as they constitute a major component of their natural diet [50]. Specimens fed with copepods reach larger sizes and produce a larger offspring. However, copepod cultivation in a scale sufficient to feed a ctenophore culture with several hundred adult animals does not only demand a considerable amount of time (requiring an additional algae culture) but also a considerable amount of space (since a too high density may lead in a crash of the culture). Frozen copepods can be an alternative. Depending on the supplier, copepods can range from 0.5 to 1.8 mm. Some aspects must be taken into account when feeding with copepods:

- Avoid thawing and freezing multiple times. To achieve this, copepods can be split into smaller volumes (e.g microcentrifuge tubes 1.5 ml or 50 ml centrifuge tubes) before freezing, and thaw only the corresponding amount needed at a time.
- Leave the frozen fraction to thaw slowly and then rinse them thoroughly with PSW using a large mesh (e.g. 500 µm).
- Copepods have to be gradually supplied to the ctenophores, particularly preventing overfeeding, since dead copepods will directly be a starter for heterotrophic bacterial growth.
- Avoid using frozen copepods on a daily basis with the described set-up. The best results can be achieved when using them the day before and the day of washing, so all the uneaten copepods will be removed after washing (Table 1).
- Water circulation in the tank has to be strong enough to keep them floating until they are captured by the ctenophores before settling down.
- After several hours, the uneaten copepods have to be removed from the bottom of the tank (see 3.2.1.2) to avoid declining water quality.
- Small ctenophores (< 5 mm) do not predate efficiently on large copepods, as they require smaller prey (see 3.2.3).
- For other recommendations, check the specifications provided by the manufacturer.
- Make sure frozen copepods do not contain any cryopreservant or other supplements, since accumulation in the ctenophores can lead to unwanted long term consequences in their health and reproduction.

##### 3.2.1.2 Washing

Clean water is fundamental to keep healthy specimens (see Note 17). Since the set-up described here does not include a continuous water exchange (as occurs with some kreisel systems with open circulation) the washing is crucial. The entire culture is washed once a week, and partial water exchange can be done additionally to ensure appropriate water quality levels (Tab. 1). Considerable variations of water parameters must be avoided to reduce the stress already caused by the washing procedure itself (see Note 7 and Note 18).

- Prepare the tanks with PSW well in advance (but see Note 6) and adjust the temperature to the existing one in the culture (see Note 5).
- If necessary, add some reverse osmosis (or deionised) water to the receiving tank in order to decrease the salinity. The contrary (i.e. increasing the salinity in the receiving tank) is not necessary unless the variation exceeds 5 ‰ (see Note 7).
- Stop, carefully remove and discard the old motion pipette.
- To transfer the specimens from a previous to a new tank, proceed as described in 3.1.2
- If evidences of damage are observed, proceed as described in Note 10.
- Install a new motion pipette and adjust it to achieve the right angle of rotation-oscillation (see Note 3).
- Rinse the old tank thoroughly with warm water and a brush. Avoid using soap or detergents.

#### 3.2.2 Breeding

**Fig. 3:**
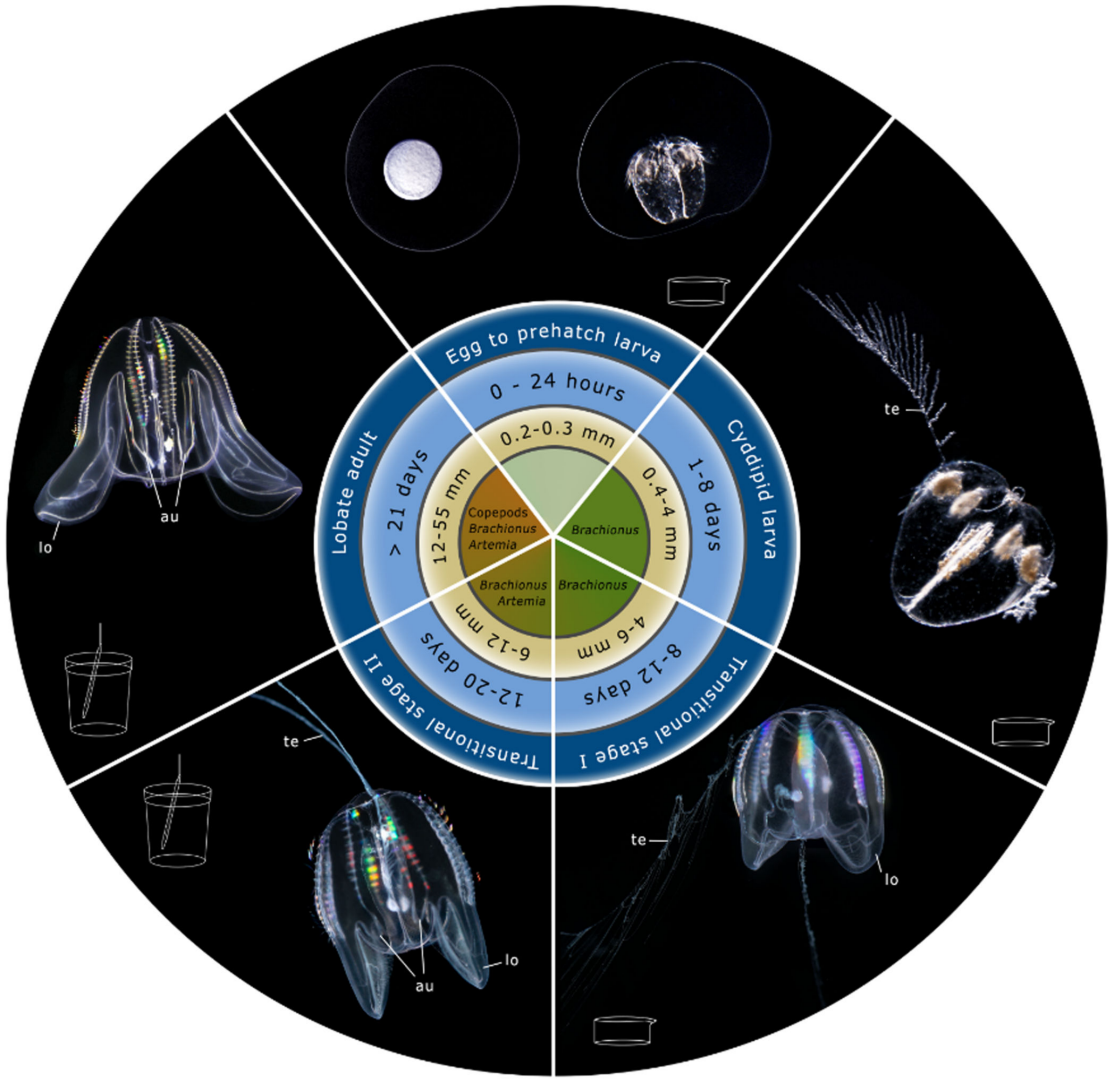
Life cycle of *Mnemiopsis leidyi* from egg to maturity including major developmental stages, time, size and main feeding prey. Transition stages are intermediate forms in which the tentacles of a cydippid larva co-occur with the lobes of a lobed adult in the same specimen. Transition stage I is characterized by the presence of early lobes until the onset of auricle development, while in transition stage II exhibits notable auricles and the lobes are clearly expanded. [**te**: tentacles; **lo**: lobes; **au**: auricles]. Figure design adapted from Ramondenc et al. 2019 [55].

Under the parameters and feeding regime described above (see section 2.1 and section 2.3), *M. leidyi* specimens reach maturity in ca. 3-4 weeks (ca. 12 mm long in oral-aboral length, see Fig. 3), with total lengths of 22 ± 9 mm after one month depending on temperature and food availability (see note 19).

**Fig. 4:**
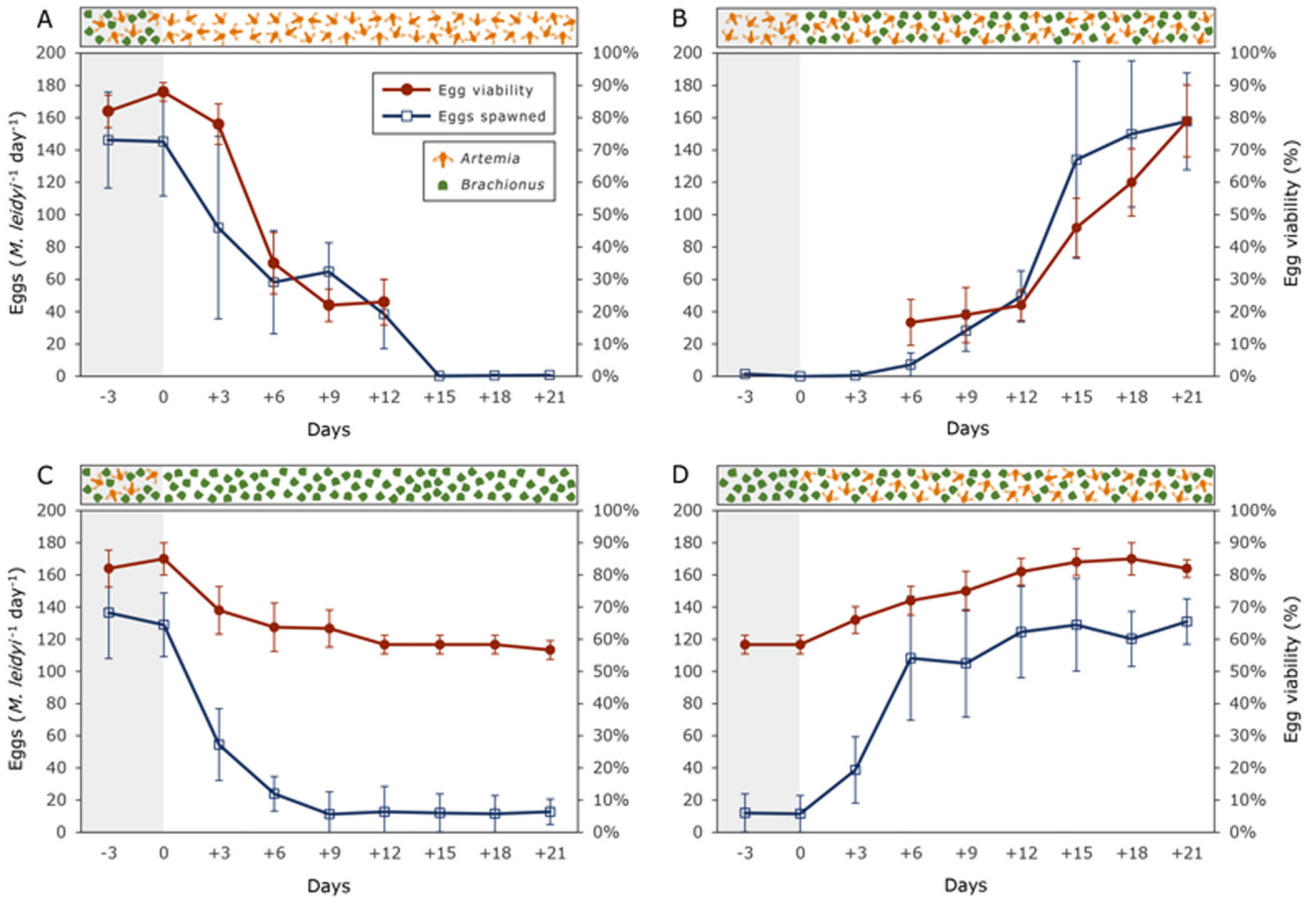
Variation in spawning intensity (number of eggs spawned per individual; n=4) and egg viability according to feeding regime. Egg viability refers to percentage of eggs that successfully developed into larvae observed at least 32 hours after fertilization (n=all spawned eggs). Note that the presence/absence of *Brachionus* or *Artemia* in the diet have dissimilar reaction time in egg production and egg viability: the effect of removing (**A**) or adding (**B**) *Brachionus* is slower than the corresponding effect of removing (**C**) or adding (**D**) *Artemia*. A diet exclusively based on *Artemia* (A) causes virtually no spawning after day 15. A period over 2-3 weeks using a combination of both prey items is therefore necessary to achieve adequate levels of spawning and high values of egg viability (B & D).

Several attempts to understand the factors inducing spawning in *M. leidyi* have been published over the last decades [26, 51]. Despite most of them coincide in the need of a timeframe of ca. 6-8 hours of complete darkness, recent contributions have shown that seasonal and population variations might condition spawning in *M. leidyi* [27] and even under a 24 h light regime, spawning can occur [52]. In addition, food conditions play an important role in reproduction rates, both at spawning level and egg viability [53]. Our experience also confirms these findings but with an interesting addition: highest spawning and egg viability rate are only achieved after a period of consecutive weeks with a varied diet (Fig. 4). In contrast, a feeding regime based exclusively on a single prey type seriously compromised reproductive success (Fig. 4). We have also observed that the spawning is not 100% synchronous, and basal spawning occurs throughout the day, at a minimum rate of 1-2 eggs per hour in healthy specimens. In general, larger ctenophores produce larger offspring [25, 52]. However, the variation is sometimes considerably wide [52] (Fig. 4), and the unpredictability requires increasing the number of replicates in order to ensure an elevated production of offspring. Taking these aspects into account, proceed as follows:

- Prepare as many 300 ml beakers as necessary at least 1 day in advance. Fill them with PSW and leave them at room temperature with a lid. This will allow to create a biofilm on the bottom of the beaker to prevent later egg adhesion (Note 20).
- Select one or two healthy (see Note 17), well-fed (Fig. 4) adults (>30 mm) and place them into a 2 L beaker filled with PSW at the same conditions than the tank where they were taken from (Note 21).
- Leave them overnight.
- The next day, check for signs of spawning. Most ctenophores spawn around 5-8 hours after programmed sunset, which can be adjusted at convenience.
- Once the spawning is complete, eggs will float during 2-4 hours before sinking. Transfer the eggs into the previously prepared 300 ml beakers while they are still floating (Note 20). Attempts of transferring eggs laying in the bottom of the beaker for several hours can result in very low survival rate (< 10 %).
- At room temperature, eggs will normally hatch 18-24 hours after fertilization [25, 26]. However, in some cases this period can be longer, up to ca. 32 hours according to our observations.

#### 3.2.3 Nursery

Once the critical hatching phase has been successfully overcome (see Note 20), the survival rate of the early hatched cydippid larvae to the adult stage under the conditions described here is very high (> 80%). The cydippid larvae (1-8 days old) and transitional stage I (8-12 days old) are raised at room temperature in 300 ml beakers (e.g., crystalizing dish) provided with a lid (e.g., the base of a tissue culture dish) to avoid water evaporation. The beakers used over the first week can be the same where the larvae hatched (but see Note 22). The nursery stage normally takes ca. 2 weeks (Fig. 3), but can be extended up to two years if necessary by regulating food availability. The only food source required during the first weeks is *Brachionus* (Fig. 3).

- As soon as the early hatched larvae are observed, feed them with *Brachionus* 4 times/week (see section 3.2.1.1.1).
- Under food saturation conditions, larvae can grow as quick as 0.5-1 mm/day (but see Note 23).
- If necessary (in case of high evaporation rates due to low humidity environment), add water every second day by pipetting PSW water to the side of the beaker to mix the water properly and allow the *Brachionus* to be evenly distributed in the beaker.
- Wash the beakers once a week by transferring the larvae to a new beaker filled with PSW (see Note 22, 24 and 25).
- Add *Brachionus* after every washing.
- After ca. 2 weeks, the specimens reach the transitional stage II, characterised by the presence of auricles, and clearly formed lobes (Fig. 3). At this point, the ctenophores can start to prey efficiently on *Artemia* nauplii, and therefore are ready to be moved to a larger tank as described in section 2.1. For transferring, proceed as described in section 3.1.2.

## 4 Notes

### Note 1

If desired, tanks can be maintained at room temperature, as long as this factor is controlled, preventing considerable circadian variations. Different *M. leidyi* populations have been successfully cultured using higher temperatures [25, 27, 51].

### Note 2

Water temperature from the tanks containing animals will only be close to the outer water in the aquarium (and therefore to the indication in the water chiller) as long as both water levels are kept as close as possible. However, beware of undesired tank flotation.

### Note 3

The flow speed can be easily adjusted by modifying pipette oscillation. The more inclined the position of the motion pipette, the stronger the water flow. Completely vertical position of the motion pipette results in only rotation, and thus very reduced current speed. Stronger flow (more inclined pipette) is adequate for smaller specimens (including eggs and larvae), whereas low current (closer to vertical position) is more appropriate for larger stages, injured and/or delicate specimens.

### Note 4

Hoses, beakers, bottles, tanks, carboys or any other device used for storage or transport water and/or move animals must be chemical-free (including soap or detergent).

### Note 5

Rinse any recipient with PSW before using, especially when they are used for transfer ctenophores or remove debris from the tanks.

### Note 6

Avoid storing seawater during several consecutive days, even PSW. Optimum water quality depends on temperature and light exposure. As a general recommendation, PSW shall be used within a week since preparation.

### Note 7

Considerable variations in water density (i.e. changes of ± 5 ‰ salinity and/or ± 5 ºC temperature) can injure the animals. In general, a water change from lower to higher salinity is stressing for the animals, resulting in partial or total disintegration. On the contrary, ctenophores adapt well from high to low salinity, even when sinking to the bottom of the recipient. If this occurs, the animal will compensate its buoyancy in approximately 1-2 hours after the water change.

### Note 8

Once the eggs hatch, and as long as the parental generation is adequately fed (see Fig. 4), *M. leidyi* larvae are particularly resistant and resilient to manipulation, water exchange, and variations in water parameters (pH, salinity and temperature). However, avoid transferring them abruptly (without a previous acclimatization period) to water temperatures under 10º C. Larvae up to a 1-2 mm long can be easily pipetted and filtered, without causing any damage.

### Note 9

Some circumstances might require transferring the least possible amount of water. These include other plankton accompanying the sample, *M. leidyi* eggs and larvae produced in the tanks, or poor water quality in the established culture due to unwanted presence of chemicals, debris, algae or bacteria. When visible, the unwanted particles or organisms can be manually removed by pipetting. If they are either too small or too abundant to be removed, proceed diluting several times before placing the ctenophore into its final destination. Note 7 also applies here.

### Note 10

*Mnemiopsis leidyi* specimens can sometimes show evidence of damage after being transferred into a new location. This does not necessarily mean that the new water is not adequate, as they might be reacting to the conditions in the previous tank or to the manipulation itself. Three scenarios can occur: 1. If more than 30% of the ctenophores in a bucket show signs of severe damage (i.e. disintegrating entire lobes, or apical region), stop the motion pipette and leave the bucket untouched until the next day. Any attempt of performing a new transfer at this point will result in further damage. The following day, transfer the whole group to a new tank and observe the response. Repeat if necessary. 2. If less than 10-15% of the ctenophores in a group show signs of severe damage, proceed as 1, but do not attempt a water exchange the next day, as the problem is not affecting the whole group and most probably not due to the new water used. In any case, monitor the state of the other ctenophores. 3. If a single (or few) ctenophore shows some signs of slight damage (e.g. in the apical section of the lobes, or a small hole in one lateral), stop the motion pipette for some hours, observe the progression, and if it looks better, switch on the motion pipette again by the end of the day. If not, wait until the next day to activate the rotation or transfer it to a separate beaker and monitor it.

### Note 11

Hundreds of larvae (<3 mm) can be kept at 6 L tanks. When specimens reach a size between ca. 10-20 mm in total length, the maximum recommended density is 5 specimens/L. For animals between 20-30 mm, a maximum density of 2-3 specimens/L is recommended.

### Note 12

Different algae products come in different concentrations, and manufacturer’s indications should be an adequate starting point. In any case, avoid overfeeding *Brachionus* culture as this might lead to crashes of the population due to high levels of ammonia and nitrite. Optimum color of the water must be slightly darker than cucumber flesh green but not as dark as cucumber peel. Daily harvesting also helps to keep appropriate water quality and a stable population over time.

### Note 13

Ideally, *Brachionus* should be harvested prior to feeding the rotifer culture, in order to reduce the amount of algae transferred from the *Brachionus* culture to the Ctenophore culture. However, harvesting starved *Brachionus* (i.e. brownish water) translates into poor-quality food. In this later case, add a small amount of algae at least one hour before harvesting.

### Note 14

Focus on the upper section of the tank containing *Brachionus* (8-9 cm). Prevent mixing during harvesting in order to avoid collecting algae precipitates laying in the bottom of the tank.

### Note 15

If necessary, place the filter into a beaker with some PSW before proceeding to rinse the harvested sample. *Brachionus* is particularly sensitive to drying out. After a few seconds of exposure to air, they become floating, thus inconvenient to be used, and a source of debris and contamination.

### Note 16

If the algae aggregates are very abundant, and/or there is a need to recover as much *Brachionus* as possible, wash off the entire *Brachionus* culture through a combination of 250 µm and 70 µm mesh. This will allow to discard most algae aggregates (250 µm mesh) while retaining *Brachionus* (70 µm mesh).

### Note 17

There are two key features that allow to categorize a *M. leidyi* specimen as healthy: when they spend most of the time with the lobes widely opened (in contrast to closed, resembling a walnut) and the auricles are clearly long and pointy, clearly surpassing the oral end (in contrast to very reduced or non-existent, and/or rounded).

### Note 18

A common practice consist of adding a large dose of food two-three hours prior to washing. Properly fed animals are less sensitive to washing procedures. However, some food should also be available after the water exchange.

### Note 19

Attempts of raising cydippid larvae into the fertile adults exclusively feeding with *Brachionus* have been satisfactory. However, the larval production at this stage is lower than in larger specimens fed with other food sources.

### Note 20

According to prior culturing attempts, manipulating and/or transferring eggs and early larvae result in very limited survival rate [25]. Certainly, egg hatching is a bottleneck in ctenophore culture. If needed, the survival rate can be improved (by a 15-20%) by transferring the eggs while still floating to the aforementioned beakers with a biofilm cover. This prevents eggs being stuck to the bottom of the recipient. Alternatively, the use of agar-coated petri dish has been described as an effective measure [26, 51].

### Note 21

Some studies have pointed out that egg viability is more reduced when a single individual self-fertilized the eggs [52, 54]. Our results do not concur in this regard, showing high viability also when spawned individually (Fig. 4).

### Note 22

Optimum conditions for nursery stage depend on the size of the specimens. Early hatched larvae can be kept by hundreds in a 300 ml beaker, but this requires a larger amount of food, with the subsequent water quality impoverishment. In addition, a large amount of larvae also leads to a slower growth rate, while the smaller densities corresponds to higher rates, up to 0.5-1 mm/day in conditions of food saturation. Our recommendation is to use maximum starting densities of 30-40 larvae in 300 ml for the first week, and split the culture the following week (15-20 larvae of 8-12 days old in 300 ml).

### Note 23

Growth rates are very different within the same offspring, resulting in ctenophores ranging from a few mm to 15 mm after the first two weeks.

### Note 24

*Mnemiopsis leidyi* eggs and larvae can be transferred using a transfer pipette. Should larvae be bigger than 2 mm, cut the tip of the transfer pipette to avoid damaging while transferring. If the tentacles are extended, induce contraction by gently touching the larva or by gently pumping water on it before pipetting. Avoid pipette transferring for lobed specimens larger than 7-8 mm, use a beaker instead.

### Note 25

*Mnemiopsis leidyi* eggs and larvae smaller than 4 mm can be transferred and/or concentrated as well by carefully filtrating through a 70 µm mesh. Avoid exposure to air by submerging the mesh into a small amount of PSW.

## Figures and Tables

**Table 1:** Overview of the weekly feeding and washing routines for *Mnemiopsis leidyi* culture. [hph: hours post-hatching].
